# Preoperative systemic inflammation response index and 12-month endoscopic recurrence after endoscopic sinus surgery in chronic rhinosinusitis with nasal polyps: association and incremental predictive performance in a same-institution, two-campus cohort

**DOI:** 10.3389/fmed.2026.1983990

**Published:** 2026-10-21

**Authors:** Xudong Qian, Shanshan Tong, Pingcun Wei, Fan Wu

**Affiliations:** 1 Department of Otorhinolaryngology—Head and Neck Surgery, Anhui No. 2 Provincial People’s Hospital (Anhui Eye Hospital), Hefei, Anhui, China; 2 Faculty of Chinese Medicine, Macau University of Science and Technology, Taipa, Macao SAR, China; 3 Department of Otorhinolaryngology—Head and Neck Surgery, The First Affiliated Hospital of Anhui Medical University, Hefei, Anhui, China

**Keywords:** calibration, chronic rhinosinusitis with nasal polyps, endoscopic sinus surgery, held-out evaluation, prediction model, recurrence, systemic inflammation response index

## Abstract

**Background:**

The systemic inflammation response index (SIRI) is a readily obtainable marker derived from routine blood count analyses and has been associated with the recurrence of chronic rhinosinusitis with nasal polyps. Whether it provides added prognostic information beyond routine clinical predictors remains uncertain.

**Methods:**

This retrospective, single-institution, two-campus study included 805 participants who were at least 18 years of age (with no upper age limit) and who underwent a endoscopic sinus surgery (ESS). The Main Campus development cohort included 445 participants, and the Huangshan Road Campus held-out cohort included 360 participants; 400 and 320 participants from both cohorts, respectively, had evaluable 12-month outcomes. Aspirin-exacerbated respiratory disease (AERD) was clinically ascertained separately from general hypersensitivity to non-steroidal anti-inflammatory drugs (NSAIDs). Clinical and extended logistic models were compared using discrimination, calibration, Brier’s score, and paired bootstrap estimates of incremental performance. Sensitivity analyses used stabilized inverse-probability weighting, 200 outcome imputations, and two extreme-outcome assignments and excluded NSAID-hypersensitive participants without AERD.

**Results:**

Recurrence occurred in 124/400 participants from the development cohort and in 99/320 participants from the held-out cohort. Each doubling of SIRI had been associated with the disease recurrence after adjustment [odds ratio, 1.47; 95% confidence interval (CI), 1.06–2.04; *P* = 0.022]. Adding SIRI changed the area under the receiver operating characteristic curve by 0.007 in the evaluation of the development cohort (bootstrap 95% CI, −0.002 to 0.026) and by 0.010 in the evaluation of the held-out cohort (−0.016 to 0.030); corresponding improvements in Brier’s score were 0.0036 (0.00006–0.0108) and 0.0044 (−0.0025 to 0.0111). Eighty-five participants lacked an evaluable outcome. The SIRI odds ratios were 1.46 with weighting, 1.47 with imputation, and 1.46 after the AERD sensitivity exclusion. Assuming that every missing outcome was recurrence attenuated the estimate to 1.31 (0.99–1.74).

**Conclusions:**

The preoperative SIRI was associated with 12-month endoscopic recurrence after ESS. This association was broadly preserved in inverse probability–weighted, multiple-imputation, and AERD-restricted analyses, although it was attenuated when all missing outcomes were assigned to recurrence. The SIRI-only model showed moderate discrimination as a simple and readily available marker of systemic inflammation, but its incremental predictive gain beyond routine clinical predictors was modest and uncertain. Therefore, these findings support SIRI as a candidate prognostic marker for prospective external validation rather than as a tool for immediate treatment or surveillance decisions.

## 1 Introduction

Chronic rhinosinusitis with nasal polyps (CRSwNP) is a heterogeneous inflammatory disorder of the nasal and paranasal sinus mucosa. Endoscopic sinus surgery (ESS) can reduce disease burden, but recurrent polyps remain an important problem during postoperative care (1, 2). Eosinophilic inflammation, asthma, aspirin-exacerbated respiratory disease (AERD), a previous surgery, and the extent of disease observed from computed tomography (CT) and endoscopy findings contribute to the recurrence risk (1–4). Recent immune-endotyping evidence further underscores this heterogeneity: Nazari et al. reported higher expression of GATA3, IL4, IL5, and IL17 in nasal polyps, together with greater infiltration of eosinophils, neutrophils, and plasma cells, in patients with AERD than in those with CRSwNP but without AERD, which is consistent with a more pronounced type 2–predominant inflammatory profile (5). These findings support careful AERD phenotyping when recurrence models are evaluated.

Clinical prediction models that incorporate peripheral blood measurements and conventional clinical characteristics have been investigated for use in CRSwNP (6). The systemic inflammation response index (SIRI), calculated from neutrophil, monocyte, and lymphocyte counts, was initially investigated as a prognostic marker in pancreatic cancer (7). A subsequent study reported an association between preoperative SIRI and postoperative CRSwNP recurrence (8). However, association alone does not establish useful added prediction. Direct evaluation of incremental performance is clinically relevant because it distinguishes prognostic association from added predictive information beyond routinely available clinical factors and defines the evidence needed for future clinical translation.

Therefore, we evaluated the association between preoperative SIRI and endoscopic recurrence by the planned 12-month visit after ESS, compared a SIRI-only model with clinical and extended models, and assessed their performance in a separate, non-overlapping cohort from a different campus of the same institution. Furthermore, we report model equations, discrimination, calibration, overall prediction error, and attrition-related uncertainty. Reporting was organized using TRIPOD+AI, which also applies to regression and machine-learning prediction models (9).

## 2 Materials and methods

### 2.1 Study design, setting, and participants

This retrospective, single-institution, two-campus study used two author-designated cohorts from Anhui No.2 Provincial People’s Hospital, Hefei, China. The Main Campus development cohort comprised 445 adults who underwent ESS between 6 January 2021 and 21 July 2024. The Huangshan Road Campus held-out cohort comprised 360 adults who underwent ESS between 1 February 2021 and 14 November 2024. The recorded follow-up cutoff was at 24 November 2025. The original campus-based allocations were retained without random redistribution. To prevent duplicate inclusion across campuses, records were crossmatched using the inpatient medical record number, national identification number, name, and date of birth; only one analytic record per patient was retained, and the final cohorts were non-overlapping. Because the surgery periods overlapped and both campuses belong to the same institution, the second cohort was treated as a different-campus, same-institution held-out evaluation cohort rather than as a temporal or external validation cohort.

An analytic extract was assembled retrospectively from routine care medical records; laboratory reports; and CT, endoscopy, surgery, pathology, and postoperative follow-up records at both campuses; no prospective research registry was used. The two campus cohorts were combined in this one analytic dataset, which was locked on 30 August 2026. After the locking of the dataset, the inclusion criteria, variable and outcome definitions, and coding rules were not changed on the basis of the observed outcomes.

Both campuses used the same eligibility criteria, extraction form, data dictionary and coding rules, SIRI calculation, AERD definition, 12-month recurrence definition, quality-control procedure, and missing-value coding, whereas their patient sources, surgery periods, sample sizes, and preassigned analytic roles differed. Both original campus analyses used the integrated locked dataset and a shared statistical framework, although the historical executable scripts could not be retrieved. The reconstructed analyses used one code base for both cohorts; development coefficients were applied unchanged in the held-out cohort.

Consecutive adults who were at least 18 years of age and had undergone ESS for CRSwNP were eligible; there was no upper age limit. CRSwNP was diagnosed in routine care using compatible clinical features together with objective nasal polyps findings on nasal endoscopy and CT imaging, consistent with EPOS 2020 (1). Additional eligibility conditions were a preoperative blood count obtained 0–7 days before surgery, no systemic glucocorticoid exposure within 14 days before blood sampling, and no recorded acute infection or fever at baseline. Participants with chronic rhinosinusitis but no nasal polyps and healthy controls were not included. The analytic extract contained the included patients only, and therefore, the total number screened and detailed counts for exclusions before inclusion were unavailable.

Participants without an evaluable planned 12-month outcome were retained in the descriptive cohort but were excluded from the primary binary-outcome model. Their last recorded follow-up or contact and reasons for non-evaluation were retained in the source data.

### 2.2 Measurements and candidate predictors

The structured data included demographics, smoking status, symptom duration, previous ESS, asthma, allergic rhinitis, general hypersensitivity to non-steroidal anti-inflammatory drugs (NSAIDs), clinically ascertained AERD, diabetes, hypertension, the 22-item Sino-Nasal Outcome Test (SNOT-22), bilateral nasal polyp score (NPS), Lund–Kennedy endoscopy score, Lund–Mackay CT score, absolute peripheral blood cell counts, C-reactive protein, total immunoglobulin E, and tissue eosinophils. General hypersensitivity to NSAIDs and AERD were abstracted as separate variables. AERD was assigned only when the clinical record documented CRSwNP and asthma together with an upper and/or lower respiratory reaction after the use of aspirin or another cyclooxygenase-1–inhibiting NSAIDs; isolated cutaneous, gastrointestinal, or non-specific reactions did not qualify. Therefore, AERD was not inferred from general hypersensitivity to NSAIDs alone. Because a study-mandated uniform provocation protocol was not used and detailed reaction phenotyping was not preserved for every participant in the analytic extract, some uncertainty inherent in retrospective clinical ascertainment remains. Tissue eosinophilia of at least 10 eosinophils per high-power field (HPF) defined eosinophilic CRS for descriptive analyses. Treatment variables were described but were not included in the prediction models.

SIRI was calculated as the absolute neutrophil count multiplied by the absolute monocyte count and then divided by the absolute lymphocyte count from the same preoperative blood sample. Counts were expressed in 10^9^ cells/L, and hence, SIRI retains that scale. Positive SIRI values were transformed using log_2_ transformation, and therefore, the corresponding odds ratio (OR) represents a doubling of SIRI. The workbook explicitly calculated SIRI with rounding to three decimal places. The primary models use that stored value to reproduce the original analysis; a calculation from the unrounded ratio was used only for the numerical check.

Authors XQ and ST jointly completed the original statistical analysis on 30 August 2026, including SIRI calculation; the SIRI-only, clinical, and extended models; and different-campus held-out evaluation. The original executable analysis code could not be recovered from the earlier analysis archive. The original principal regression analyses used neither random splitting nor random resampling, and hence, no random seed applied to those fits. For this revision, author XQ reconstructed the primary calculations and development model coefficients. Author ST independently checked the models, applied the unchanged coefficients in the held-out cohort, recalculated performance, and regenerated the receiver operating characteristic (ROC) and calibration figures. Authors ST and FW performed the additional bootstrap, missing-outcome, and AERD sensitivity analyses. Author PW verified the clinical source records, eligibility and variable definitions, surgery data, and recurrence outcomes. Author FW reviewed model specification, supervised the revised analyses, and confirmed the final results. The reconstruction and figure code and its applicable random seeds were retained separately from the unavailable original code (Supplementary Methods S6).

The clinical model included eight predictors specified in the source analysis: age, sex, asthma, AERD, the number of previous ESS procedures, the Lund–Mackay CT score, baseline total NPS, and the absolute blood eosinophil count. The extended model added log_2_(SIRI). Continuous predictors were retained continuously without outcome-derived dichotomization or univariable *P*-value selection. The development analysis contained 124 events and nine predictor coefficients in the extended model, corresponding to 13.8 events per predictor coefficient. This descriptive ratio is not a formal sample-size justification; no prospective calculation based on the anticipated model performance or shrinkage was documented (10).

The eight-predictor clinical model was retained as the fixed benchmark from the source analysis for assessing the incremental contribution of SIRI. The symptom duration, SNOT-22, Lund–Kennedy endoscopy score, and C-reactive protein and total immunoglobulin E levels were not added on the basis of their descriptive associations with recurrence in Table 1.

**Table 1:** Development cohort characteristics by recurrence status.

Variable	No recurrence (*n* = 276)	Recurrence (*n* = 124)	*P-*value
Age, years	46.1 ± 13.2	47.5 ± 14.1	0.351
Male sex	139 (50.4)	78 (62.9)	0.020
BMI, kg/m^2^	24.6 ± 3.2	24.8 ± 3.3	0.426
Smoking	57 (20.7)	25 (20.2)	0.910
Symptom duration, months	43 (28, 68)	58 (44, 97)	<0.001
Previous ESS, procedures	0 (0, 1)	0 (0, 1)	0.261
Revision ESS	72 (26.1)	40 (32.3)	0.204
Asthma	69 (25.0)	48 (38.7)	0.005
Allergic rhinitis	112 (40.6)	61 (49.2)	0.108
NSAID hypersensitivity	13 (4.7)	19 (15.3)	<0.001
AERD	8 (2.9)	15 (12.1)	<0.001
Baseline SNOT-22	44.8 ± 12.1	51.2 ± 12.3	<0.001
Baseline NPS	4.3 ± 1.5	5.2 ± 1.5	<0.001
Baseline Lund–Kennedy score	8.9 ± 2.3	10.2 ± 2.1	<0.001
Lund-Mackay CT score	11.6 ± 4.5	15.1 ± 4.0	<0.001
Blood eosinophils, ×10^9^ cells/L	0.21 (0.15, 0.30)	0.29 (0.22, 0.42)	<0.001
CRP, mg/L	1.98 (1.41, 2.88)	2.59 (1.80, 4.22)	<0.001
Total IgE, IU/mL	162 (95, 240)	212 (126, 416)	<0.001
SIRI	0.847 (0.569, 1.232)	1.367 (0.845, 2.095)	<0.001
Eosinophilic CRS (≥10 cells/HPF)	190 (70.1)	118 (95.9)	<0.001

Values are expressed as mean ± SD, median (25th, 75th percentile), or *n* (%). Percentages use non-missing denominators. Two-sided Welch’s t-tests, Mann-Whitney U tests, or Pearson chi-square tests (Fisher exact when expected counts <5) were performed, as appropriate. These descriptive comparisons were not used for predictor selection; *P*-values are unadjusted for multiple comparisons.

BMI, body mass index; CRP, C-reactive protein.

Absolute neutrophil, monocyte, lymphocyte, and eosinophil counts were obtained from the same routine preoperative complete blood count in the hospital clinical laboratory under the standard operating and internal quality-control procedures in effect at the time of testing. Baseline CT, nasal endoscopy, and histopathology variables were abstracted from routine care records. CT burden was recorded using the Lund–Mackay score, endoscopic burden using the total bilateral NPS and Lund–Kennedy score, and eosinophilic CRS as at least 10 tissue eosinophils per high-power field for descriptive analyses. The final values in the signed clinical records were used. When duplicate structured entries differed, the underlying report was reviewed and discrepancies were resolved by consensus before the database was locked; unresolved values were treated as missing without reference to the recurrence status. Original images and specimens were not reread specifically for this study, and interrater agreement was not estimated.

### 2.3 Outcome and follow-up

The primary outcome was endoscopic polyp recurrence by the planned postoperative 12-month assessment, which was defined as NPS ≥1 on either side. Longitudinal records included scheduled visits at approximately 3, 6, and 12 months and additional recurrence assessments for some participants. The actual planned 12-month visit window was 11.3–13.0 months after ESS; accordingly, the endpoint is visit-based rather than an exact event risk at postoperative day 365. Earlier recorded recurrence contributed to the binary endpoint. First recurrence dates and ascertainment modes were descriptive; no time-to-event model was fitted.

The outcome status was evaluable in 400 development and 320 held-out participants; 45 and 40 participants, respectively, had no evaluable outcome. The primary outcome was reappearance of endoscopically visible polyps after documented postoperative clearance, which was defined as NPS ≥1 on either side by the planned 12-month assessment. The clinicians who were documenting routine follow-up endoscopy were not provided with the study-calculated SIRI value or the model-derived risk estimate. Because the model was developed retrospectively, its predictions were not available during outcome assessment; no claim was made that assessors were masked to the remainder of the clinical record.

### 2.4 Model development and performance assessment

Three unpenalized binary logistic regression models were fitted in the 400 evaluable development participants: a SIRI-only model using log_2_(SIRI), the eight-predictor clinical model, and the extended model adding log_2_(SIRI). Age was modeled for every 10 years, the Lund–Mackay CT score for every 5 points, and blood eosinophils for every 0.1 × 10^9^ cells/L. Male sex, asthma, and AERD were coded as 1 when present and as 0 otherwise. Continuous effects were modeled linearly on the log-odds scale apart from log_2_(SIRI); non-linear effects were not evaluated. Multicollinearity was assessed using variance inflation factors. The equation is the definitive model specification, and no bedside decision tool is proposed.

Development intercepts and coefficients were applied unchanged to the held-out evaluation cohort, with no predictor reselection, coefficient updating, or recalibration. Internal validation used 1,000 non-parametric bootstrap resamples of the development cohort, repeating model estimation and comparing performance in each resample with that in the original development sample. Apparent and optimism-corrected performances are reported separately. The reproducible verification procedure is described in the Supplementary Material.

Discrimination was assessed using the area under the receiver operating characteristic curve (AUC) with 95% confidence intervals (CIs) for each model. The overall prediction error was measured with the Brier score. Calibration-in-the-large was estimated using a logistic model with the original linear predictor as an offset; a separate calibration model estimated an intercept and slope. Ideal calibration-in-the-large is 0, and the ideal slope is 1 (11, 12). Apparent development calibration is inherently optimistic for an unpenalized fitted model and was not interpreted as independent validation.

Model-specific AUC 95% CIs used DeLong's variance estimator, but no DeLong hypothesis test was used to compare the nested clinical and extended models. Incremental performance was quantified as the paired change in AUC (extended − clinical) and improvement in Brier’s score (clinical − extended). Uncertainty was estimated using 2,000 paired non-parametric bootstrap replicates. Each replicate independently resampled participants of the development and held-out cohorts, refitted both models in the development resample, and evaluated paired predictions in the development and held-out resamples; 95% CIs used the 2.5th and 97.5th percentiles of the bootstrap distribution. The likelihood ratio test assessed additional association and model fit in development, and its results were not interpreted as evidence of clinical utility (13). Reclassification indices and decision-curve analysis were not used because no specific clinical action, externally justified risk threshold, or utility weighting had been prespecified.

### 2.5 Missing-outcome and AERD sensitivity analyses

The primary analysis included participants with evaluable 12-month outcomes. Potential attrition bias was examined using standardized mean differences for all model predictors and cohorts and by comparing fixed-model predicted recurrence risks between participants with and without evaluable outcomes. An observation probability model used cohort and all nine extended-model predictors to derive untruncated stabilized inverse-probability-of-observation weights; weighted regression used a robust sandwich covariance. In addition, missing binary outcomes were addressed using 200 proper stochastic logistic imputations performed separately by cohort under a missing-at-random assumption. The imputation model contained all nine predictors, propagated coefficient uncertainty via multivariate normal draws, and generated outcomes through Bernoulli draws. SIRI coefficients were pooled with Rubin’s rules.

Two deterministic extreme scenarios assigned first all 85 missing outcomes to no recurrence and then to recurrence, followed by model refitting. To distinguish AERD from other NSAID reactions, a separate analysis excluded all 25 participants with recorded NSAID hypersensitivity but without clinically ascertained AERD, including 23 with evaluable outcomes, and refitted the clinical and extended models while retaining every participant with AERD.

### 2.6 Statistical analysis and reproducibility

Continuous variables are expressed as mean ± SD or as median (first quartile, third quartile). Between-group comparisons used two-sided Welch’s t-tests or Mann–Whitney’s U tests, as appropriate. Categorical variables are reported as *n* (%) and were compared using Pearson chi-square tests without continuity correction or using Fisher’s exact tests when expected counts were small. Standardized mean differences, rather than significance tests alone, were used to describe imbalance by endpoint availability. Tests were two-sided with alpha = 0.05; descriptive *P*-values were not adjusted for multiplicity and were not used for predictor selection.

All primary predictors were available for all 805 participants. The primary available-outcome analyses included 720 participants overall (400 from development and 320 from held-out cohorts). Model fitting used only the 400 development cohort participants, and the performance of the held-out cohort was evaluated in the 320 participants using unchanged development coefficients. Missing outcomes were not coded as non-recurrence. Other measurements were summarized using available observations. Missing-outcome weighting, imputation, and extreme assignments were conducted as sensitivity analyses and were not substituted for the primary estimates.

Statistical verification was performed in Python 3.12.14 using NumPy 2.3.5, pandas 2.2.3, SciPy 1.17.0, and scikit-learn 1.8.0. Internal validation of the extended model used 1,000 resamples (seed 20260830), paired refit-bootstrap intervals used 2,000 successful resamples (seed 20260917), and outcome imputation used seed 20260918. Full computational details are provided in the Supplementary Material.

## 3 Results

### 3.1 Participant flow and cohort characteristics

The dataset contained 805 included participants: 445 in the development cohort and 360 in the held-out evaluation cohort. Outcomes were evaluable in 720/805 (89.4%) participants; 85/805 (10.6%) lacked an evaluable endpoint. Recurrence occurred in 124/400 participants from the development cohort (31.0%) and 99/320 participants from the held-out cohort (30.9%) for a total of 223/720 (31.0%). Participants without an evaluable endpoint were younger than participants with an evaluable endpoint (41.6 ± 11.5 vs. 47.7 ± 13.0 years; absolute standardized mean difference, 0.499; *P* < 0.001). Absolute standardized mean differences for the other model predictors were at most 0.141. Mean fixed extended-model predicted risk was 0.312 among participants without an endpoint and was 0.320 among evaluable participants (standardized mean difference, 0.039; *P* = 0.738). In the observation model, older age was associated with endpoint availability (OR per 10 years, 1.48; 95% CI, 1.23–1.79; *P* < 0.001), whereas log_2_(SIRI) was not (OR, 1.02; 95% CI, 0.75–1.40; *P* = 0.885). These measured variable comparisons do not establish that missingness was non-informative. Participants without an endpoint had a median recorded follow-up of 5.09 months [interquartile range (IQR), 3.71–7.82]; Figure 1 and Supplementary Table S2 report the recorded reasons for loss.

**Figure 1:**
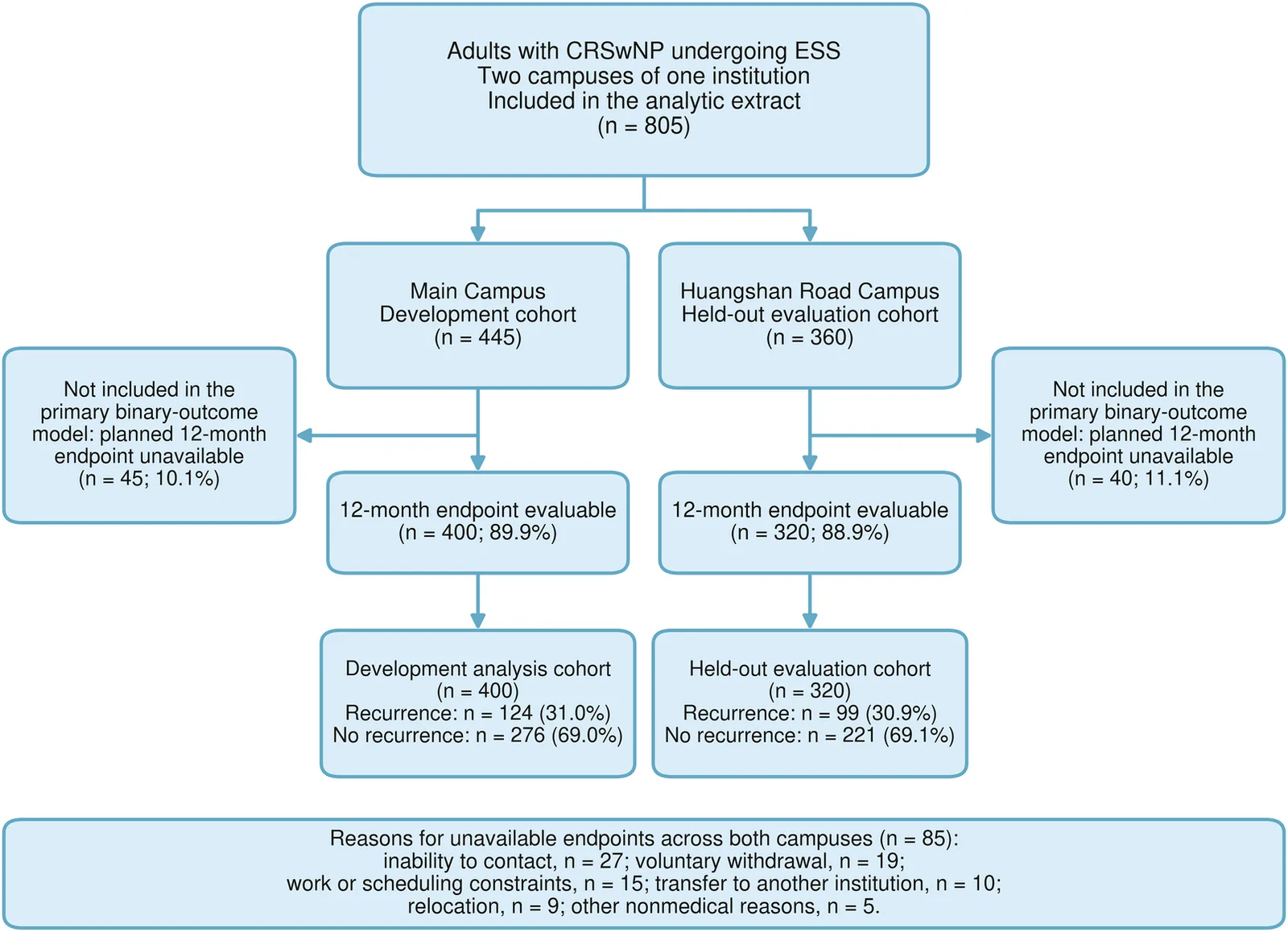
A participant flow diagram and availability of the planned 12-month endoscopic recurrence endpoint. The analytic extract included 805 adults with CRSwNP who underwent ESS at two campuses of the same institution. The Main Campus development cohort contained 445 participants; 45 lacked an evaluable planned 12-month endpoint and 400 entered model fitting. The Huangshan Road Campus held-out cohort contained 360 participants; 40 lacked an evaluable endpoint and 320 entered held-out evaluation using unchanged development coefficients. Recurrence occurred in 124/400 and 99/320 evaluable participants, respectively. Across both campuses, recorded reasons for the 85 unavailable endpoints were inability to contact (27), voluntary withdrawal (19), work or scheduling constraints (15), transfer to another institution (10), relocation (9), and other non-medical reasons (5). The analytic extract contained only included participants, and therefore, the total number screened and counts excluded before inclusion were unavailable.

Baseline characteristics are summarized in Table 2. Participants from the held-out cohort were older on average and had a longer reported symptom duration. The observed recurrence proportions were similar (*P* = 0.986). The full included cohorts, including participants with unavailable 12-month outcomes, were used for baseline descriptions; model performance uses only the evaluable cohorts.

**Table 2:** Characteristics of the included cohorts.

Variable	Development (*n* = 445)	Held-out evaluation (*n* = 360)	*P-*value
Age, years	46.1 ± 13.4	48.3 ± 12.3	0.017
Male sex	242 (54.4)	207 (57.5)	0.376
BMI, kg/m^2^	24.6 ± 3.3	24.9 ± 3.5	0.388
Smoking	98 (22.0)	77 (21.4)	0.828
Symptom duration, months	49 (32, 75)	55 (35, 88)	0.010
Revision ESS	125 (28.1)	107 (29.7)	0.611
Asthma	135 (30.3)	120 (33.3)	0.364
Allergic rhinitis	197 (44.3)	160 (44.4)	0.960
AERD	26 (5.8)	18 (5.0)	0.601
Baseline SNOT-22	46.9 ± 12.6	48.1 ± 12.0	0.171
Baseline NPS	4.6 ± 1.5	4.8 ± 1.5	0.051
Baseline Lund–Kennedy score	9.4 ± 2.3	9.6 ± 2.4	0.110
Lund-Mackay CT score	12.7 ± 4.6	12.9 ± 4.7	0.576
Blood eosinophils, ×10^9^ cells/L	0.24 (0.16, 0.34)	0.25 (0.17, 0.37)	0.304
CRP, mg/L	2.15 (1.49, 3.26)	2.19 (1.47, 3.34)	0.764
Total IgE, IU/mL	179 (101, 286)	170 (91, 323)	0.753
SIRI	1.005 (0.607, 1.512)	1.006 (0.653, 1.596)	0.360
Eosinophilic CRS (≥10 cells/HPF)	346 (78.8)	272 (78.2)	0.824
12-month outcome evaluable	400 (89.9)	320 (88.9)	0.647
Recurrence among patients with evaluable outcomes	124 (31.0)	99 (30.9)	0.986

Values are expressed as mean ± SD, median (25th, 75th percentile), or *n* (%). Percentages use non-missing denominators. AERD was clinically ascertained separately from general hypersensitivity to NSAIDs. Two-sided descriptive tests were not used for predictor selection; *P*-values are unadjusted for multiple comparisons. Missing overall: baseline SNOT-22, 7; C-reactive protein, 18; total immunoglobulin E, 64; and histological classification, 18.

### 3.2 Characteristics by recurrence status

Among development participants with evaluable outcomes, recurrence was associated in descriptive comparisons with a higher proportion of men, asthma, hypersensitivity to NSAIDs, and AERD; longer symptom duration; higher baseline SNOT-22, NPS, Lund–Kennedy, and Lund–Mackay CT scores; higher blood eosinophil count; C-reactive protein and total immunoglobulin E levels; and higher SIRI (Table 1). The median SIRI was 1.367 (0.845, 2.095) in participants with recurrence and 0.847 (0.569, 1.232) in those without recurrence (*P* < 0.001). The direction was consistent in the held-out cohort: 1.512 (0.975, 2.158) vs. 0.842 (0.587, 1.315), respectively (*P* < 0.001).

### 3.3 Regression estimates and model specification

In the extended development model, each doubling of SIRI remained associated with higher odds of recurrence (OR, 1.47; 95% CI, 1.06–2.04; *P* = 0.022). The OR per 5-point increase in Lund–Mackay CT score was 1.73 (95% CI, 1.23–2.44; *P* = 0.002). The remaining predictors did not meet the nominal significance threshold (Table 3). AERD had an imprecise adjusted estimate (OR, 1.78; 95% CI, 0.60–5.28; *P* = 0.302), which should not be interpreted as evidence of no association. After excluding participants with NSAID hypersensitivity but without clinically ascertained AERD, the SIRI estimate was 1.46 (95% CI, 1.05–2.04; *P* = 0.026), and the AERD estimate was 1.80 (0.60–5.35; *P* = 0.293).

**Table 3:** Univariable and multivariable logistic regression in the development cohort.

Predictor	Univariable OR (95% CI)	*P-*value	Adjusted OR (95% CI)	*P-*value
Age (per 10 years)	1.08 (0.92–1.27)	0.337	1.12 (0.94–1.34)	0.199
Male sex	1.67 (1.08–2.58)	0.020	1.42 (0.88–2.30)	0.154
Asthma	1.89 (1.21–2.98)	0.006	0.94 (0.53–1.69)	0.841
AERD	4.61 (1.90–11.19)	<0.001	1.78 (0.60–5.28)	0.302
Previous ESS (per procedure)	1.15 (0.82–1.62)	0.413	0.81 (0.54–1.22)	0.313
Lund-Mackay CT score (per 5 points)	2.50 (1.90–3.30)	<0.001	1.73 (1.23–2.44)	0.002
Baseline NPS (per point)	1.50 (1.29–1.74)	<0.001	1.17 (0.98–1.40)	0.087
Blood eosinophils (per 0.1 × 10^9^ cells/L)	1.38 (1.21–1.56)	<0.001	1.14 (0.97–1.34)	0.110
SIRI (per doubling)	2.33 (1.78–3.04)	<0.001	1.47 (1.06–2.04)	0.022

OR, odds ratio; CI, Wald confidence interval.

The extended model simultaneously included all nine listed predictors (400 participants; 124 events). AERD was clinically ascertained and was distinct from general NSAID hypersensitivity. Binary predictors: male/yes = 1, female/no = 0. No univariable screening was used.

The extended-model LP, with coefficients displayed in up to four decimal places, is

$$\begin{array}{rl} \mathrm{LP}= & -4.1085+0.1153\times (\mathrm{age}/10)+0.3503\times \mathrm{male}-0.0599\\ & \times \mathrm{asthma}+0.5744\times \mathrm{AERD}-0.2094\times \mathrm{previous}\;\mathrm{ESS}\;\mathrm{count}\\ & +0.5495\times (\mathrm{Lund}\text{–}\mathrm{Mackay}\;\mathrm{CT}/5)+0.1561\times \mathrm{baseline}\;\mathrm{NPS}\\ & +0.1320\times (\mathrm{blood}\;\mathrm{eosinophils}/0.1)+0.3839\times \mathrm{lo}g_{2}(\mathrm{SIRI}) \end{array}$$

Predictedprobability=1/[1+exp(−LP)].

Age is in years, and blood eosinophil count is in 10^9^ cells/L. Male sex, asthma, and AERD are binary indicators. Supplementary Table S1 supplies full-precision coefficients and coding.

### 3.4 Discrimination, calibration, and incremental performance

The SIRI-only, clinical, and extended models had development AUCs of 0.708 (95% CI, 0.652–0.763), 0.757 (0.709–0.805), and 0.764 (0.716–0.812), respectively, and their corresponding Brier scores were 0.189, 0.179, and 0.175. A reproducible 1,000-resample verification bootstrap gave an optimism-corrected AUC of 0.740, a corrected Brier score of 0.185, and a mean test-sample calibration slope of 0.874. Complete resampling details for these internal validation results are reported in the Supplementary Material.

When the development coefficients were applied unchanged in the held-out cohort, the SIRI-only, clinical, and extended models had AUCs of 0.726, 0.781, and 0.791, respectively (Table 4, Figure 2). The extended model had a held-out Brier score of 0.164, a calibration-in-the-large of −0.125 (95% CI, −0.388 to 0.138), and a calibration slope of 1.185 (95% CI, 0.869–1.502; Figure 3). These estimates indicate some calibration deviation but are imprecise.

**Table 4:** Model performance in development and held-out evaluation.

Cohort	Model	AUC (95% CI)	Brier’s score	Calibration-in-the-large	Calibration slope
Development cohort	SIRI-only	0.708 (0.652–0.763)	0.189	0 (apparent)	1 (apparent)
Development cohort	Clinical	0.757 (0.709–0.805)	0.179	0 (apparent)	1 (apparent)
Development cohort	Extended	0.764 (0.716–0.812)	0.175	0 (apparent)	1 (apparent)
Held-out evaluation cohort	SIRI-only	0.726 (0.666–0.786)	0.182	−0.053	1.183
Held-out evaluation cohort	Clinical	0.781 (0.728–0.835)	0.168	−0.135	1.141
Held-out evaluation cohort	Extended	0.791 (0.737–0.844)	0.164	−0.125	1.185

AUC 95% CIs are model-specific DeLong intervals; no DeLong test was used to compare the nested models. Development values are apparent; held-out values use unchanged development coefficients. Paired refit-bootstrap estimates are given as follows: a development delta AUC of 0.007 (95% CI, −0.002 to 0.026) and an improvement in Brier’s score of 0.0036 (0.00006–0.0108); a held-out delta AUC of 0.010 (−0.016 to 0.030) and an improvement in Brier’s score of 0.0044 (−0.0025 to 0.0111). Differences use unrounded values. Internal validation is reported in Supplementary Table S7.

**Figure 2:**
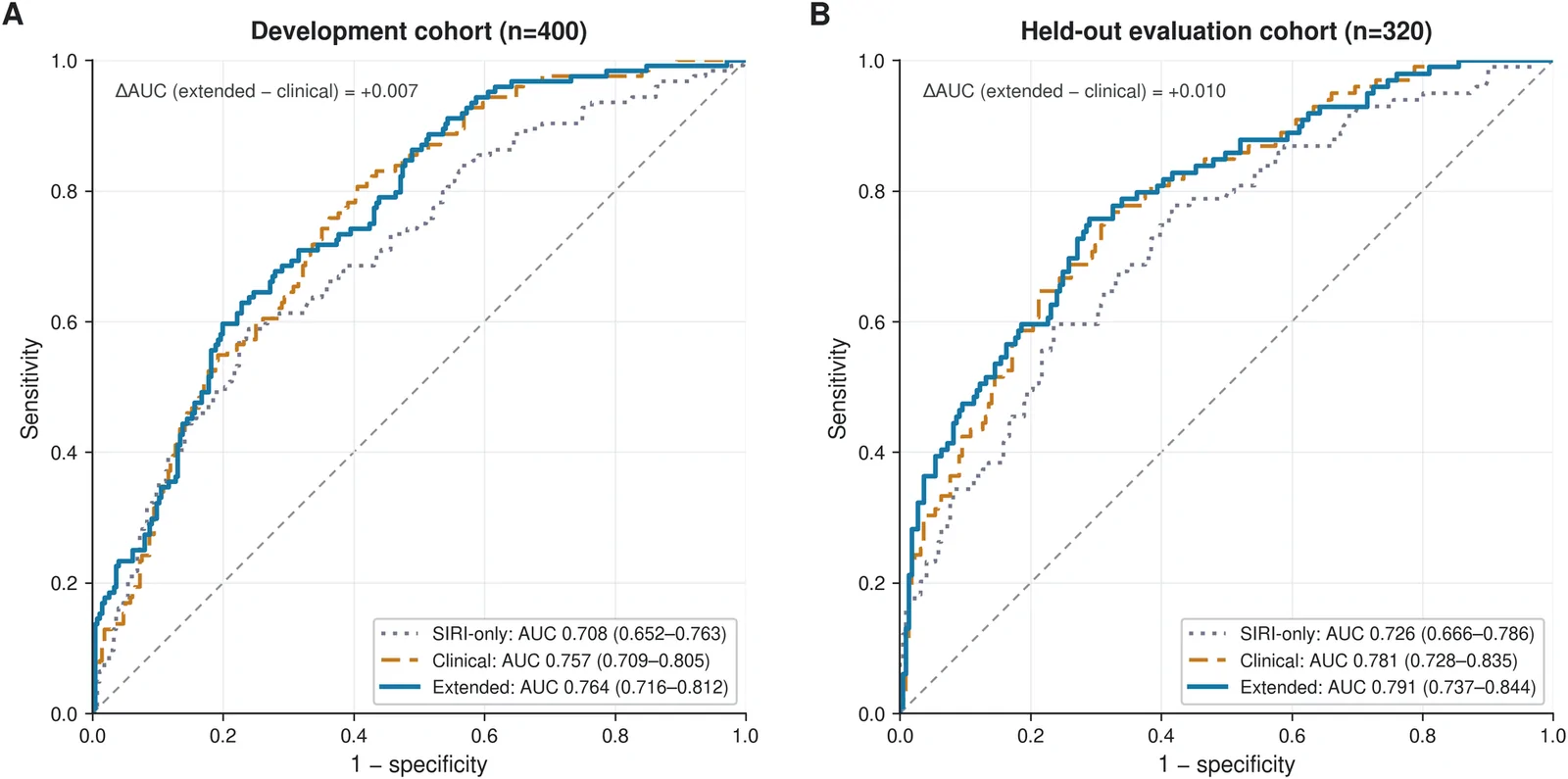
Receiver operating characteristic curves. **(A)** Apparent development performance (*n* = 400). **(B)** Held-out evaluation performance with unchanged development coefficients (*n* = 320). Curves compare SIRI-only, clinical, and extended models. Incremental AUCs for the extended model vs. clinical model were 0.007 (paired refit-bootstrap 95% CI, −0.002 to 0.026) in the evaluation of the development cohort and 0.010 (−0.016 to 0.030) in the evaluation of the held-out cohort. No hypothesis test of the nested-model AUC difference was used.

**Figure 3:**
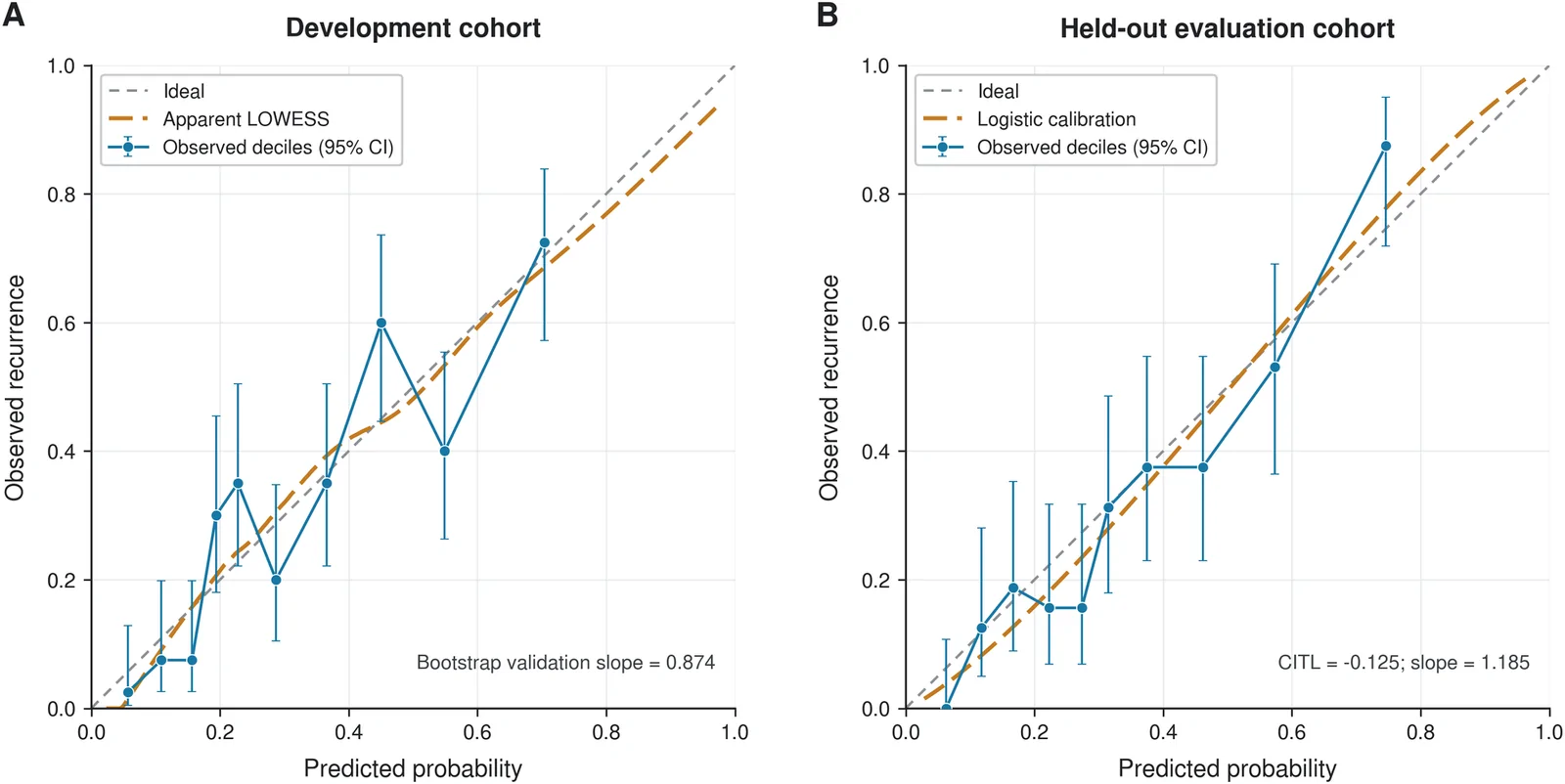
Calibration plots. **(A,B)** Observed recurrence with Wilson 95% CIs and mean prediction in 10 quantile groups for the evaluation of development and held-out cohorts, respectively. Development LOWESS uses frac = 2/3 and it = 0. The held-out logistic calibration curve uses a jointly fitted intercept and slope; calibration-in-the-large is estimated separately with slope fixed to 1. The development bootstrap annotation is the mean original-sample test calibration slope from 1,000 refits. No decision curve or clinical utility claim is made.

All three AUC point estimates were higher in the held-out cohort than in the development cohort (SIRI-only, 0.726 vs. 0.708; clinical, 0.781 vs. 0.757; and extended, 0.791 vs. 0.764). All three calibration slopes of the held-out cohort exceeded 1 (Table 4), whereas the mean bootstrap test slope for the development extended model was 0.874.

Adding SIRI changed the AUC by 0.007 in the evaluation of the development cohort (paired refit-bootstrap 95% CI, −0.002 to 0.026) and by 0.010 in the evaluation of the held-out cohort (−0.016 to 0.030). Brier score improvements were 0.0036 (95% CI, 0.00006–0.0108) and 0.0044 (−0.0025 to 0.0111), respectively. Point estimates favored the extended model, but both AUC intervals and the held-out Brier interval included zero, and therefore, any incremental gain was modest and imprecisely estimated and did not establish clinically meaningful improvement. The likelihood ratio test of the development cohort supported additional model-fit information (chi-square = 5.37, 1 degree of freedom, *P* = 0.020) but was not interpreted as evidence of clinical usefulness.

### 3.5 Sensitivity analyses

Stabilized inverse-probability weighting produced a SIRI OR of 1.46 per doubling (robust 95% CI, 1.03–2.08; *P* = 0.034), and the 200-imputation Rubin-pooled estimate was 1.47 (95% CI, 1.06–2.05; *P* = 0.021). Assigning every missing outcome to no recurrence gave an OR of 1.44 (95% CI, 1.05–1.98; *P* = 0.022). Assigning every missing outcome to recurrence attenuated the estimate to 1.31 (95% CI, 0.99–1.74; *P* = 0.062); the direction remained positive, but statistical significance was not retained under this deliberately extreme assumption. These assignments bounded the overall recurrence proportion at 223/805 (27.7%) and 308/805 (38.3%), respectively. After exclusion of the 25 participants with hypersensitivity to NSAIDs but without AERD, 391 and 306 participants with evaluable outcomes from the development and held-out cohorts, respectively, remained. The adjusted SIRI OR was 1.46 (95% CI, 1.05–2.04; *P* = 0.026), and held-out clinical and extended AUCs were 0.7817 and 0.7893, respectively (difference, 0.0076; based on unrounded estimates). These analyses evaluate robustness under specified assumptions but cannot establish the unobserved recurrence status of participants lost to follow-up (Supplementary Tables S3–S5).

## 4 Discussion

In this same-institution, two-campus cohort, higher preoperative SIRI remained associated with 12-month endoscopic recurrence after multivariable adjustment. The estimates were similar in inverse probability–weighted, multiple-imputation, and AERD-restricted analyses, and the direction remained positive under both extreme missing-outcome assignments, although it was attenuated when every missing outcome was assigned to recurrence. The SIRI-only model showed moderate discrimination in both cohorts. Adding SIRI to routine clinical predictors produced small numerical improvements in AUC and Brier’s score, although the paired bootstrap intervals were compatible with no incremental gain. Together, these findings identify SIRI as a readily available candidate prognostic marker while showing that additional validation and clinically defined thresholds are needed before it can guide management.

The results should be interpreted within the prediction-model bias domains emphasized by PROBAST, particularly participant selection, outcome assessment, and analysis (14). Previous studies have connected eosinophilic inflammation and clinical disease burden with recurrence (3, 4), and SIRI has previously been evaluated in CRSwNP (8). Other models have combined routinely available clinical and laboratory measurements (6, 15). The present analysis extends this literature by directly benchmarking SIRI against a clinical model, evaluating calibration and overall prediction error, applying unchanged coefficients in a non-overlapping different-campus cohort, and examining attrition-related uncertainty. Therefore, it quantifies both the adjusted prognostic association of SIRI and the current magnitude and uncertainty of its incremental predictive contribution. Because the held-out cohort remained within the same institutional system, transportability across independent hospitals still requires evaluation.

SIRI combines cell counts that can reflect systemic inflammatory and immune status (7). A relationship with recurrence is biologically plausible, but the present observations cannot establish that SIRI measures a distinct causal pathway or a specific CRSwNP endotype. Infection, medication exposure, and comorbidity can affect its components. The recorded restrictions on blood sampling and recent glucocorticoids address some sources of variation, but unmeasured influences remain possible. The adjusted OR of 1.47 describes higher odds per doubling but not a 47% increase in absolute recurrence risk.

Statistical association and incremental prediction answer different questions. A predictor can improve likelihood-based model fit without materially changing the ordering or absolute accuracy of individual risks (11–13). Here, the likelihood ratio test supported added association, whereas the AUC and Brier changes were small. Therefore, the results did not demonstrate material improvement in individual risk classification or patient management.

Because SIRI is calculated from a routine complete blood count analysis, it remains a readily obtainable candidate for further prognostic evaluation. Future prospective studies should validate the findings in independent institutions; minimize outcome loss; and prespecify the intended clinical action, corresponding threshold, and utility weighting. Until such evidence is available, SIRI should not be used alone to alter endoscopic surveillance, biologic therapy, postoperative anti-inflammatory treatment, or revision-surgery decisions.

Overall, the strengths of this study include explicit comparison with a clinical model, retention of continuous predictors, complete primary predictor data, application of unchanged coefficients to a separate cohort, and disclosure of the model equation and multiple performance measures. The analysis did not use univariable screening to determine the final predictor set. Nevertheless, retrospective designation of predictors as prespecified requires corroboration from a dated analysis plan; transparent reporting does not itself establish a low risk of bias (9, 14).

However, several limitations constrain interpretation. First, the cohorts came from different campuses but remained under one institutional system, and their surgery periods overlapped. All three held-out AUC point estimates were higher than their apparent development counterparts, whereas calibration slopes above 1 indicate deviation from ideal calibration rather than superior calibration. The optimism-corrected development AUC of 0.740 and the held-out extended-model AUC of 0.791 arise from different evaluation procedures, and their difference cannot identify a cause. Sampling variation or campus differences in case mix, treatment, or outcome ascertainment may contribute. The held-out extended-model slope was imprecise (1.185; 95% CI, 0.869–1.502). Same-institution evaluation does not establish transportability to independent hospitals. Second, 85 participants (10.6%) lacked evaluable outcomes and were younger than participants with evaluable outcomes. Stabilized weighting, multiple imputation, predicted-risk comparison, and extreme-outcome refits broadened the assessment beyond complete cases. The weighting and imputation estimates were close to the primary estimate, whereas the all-missing-recurrence scenario included the null. None of these analyses eliminates selection bias because missing-at-random assumptions are untestable and the extreme scenarios are deliberately unrealistic bounds. Third, the 11.3–13.0-month visit window and intermittent endoscopy introduce variability in ascertainment time; the binary model does not estimate an exact day-365 risk or address interval censoring.

Fourth, retrospective clinical records may contain residual confounding and incomplete treatment information. AERD was clinically ascertained from respiratory NSAID reactions in participants with asthma and CRSwNP and was not inferred from general NSAID hypersensitivity. However, retrospective ascertainment without a uniform study-mandated aspirin challenge or complete reaction-phenotype documentation leaves potential for residual misclassification. Excluding participants with hypersensitivity to NSAIDs but without clinically ascertained AERD did not materially change the SIRI estimate (OR per doubling, 1.46; 95% CI, 1.05–2.04), indicating that classification of this separate NSAID hypersensitivity group did not explain the observed SIRI association. Nevertheless, residual misclassification within the clinically ascertained AERD group remains possible. Overall, only 44 participants had AERD, including 23 in the development analysis. Therefore, the adjusted AERD estimate was imprecise, and its non-significance should not be interpreted as evidence of no AERD effect. Fifth, no information on formal sample-size calculation, prospective registration, subgroup performance analysis, or clinical impact evaluation was supplied. Sixth, calibration and incremental performance intervals were wide, particularly in the evaluation of held-out cohorts.

The extended model’s mean bootstrap test calibration slope of 0.874 was below the 0.90 global-shrinkage target used in prediction model sample-size planning (10), suggesting some overfitting of the unpenalized coefficients. No penalized or uniformly shrunken model with an updated intercept was fitted. Therefore, the displayed equation and its absolute risk predictions require further validation and assessment of recalibration or shrinkage before clinical use. The fixed eight-predictor comparator also omitted other measured candidate predictors, including C-reactive protein, which may share inflammatory information with SIRI. The estimated incremental performance is conditional on this comparator.

No hypothesis test *P-*value is reported for the nested-model AUC difference. Paired refit-bootstrap intervals describe uncertainty in the AUC and Brier score changes, while the likelihood ratio test addresses only added association and fit (13). Decision-curve analysis was not performed because the study did not specify a clinical action or defensible action threshold. Further work should prospectively validate the model in independent institutions, minimize outcome loss, and define a specific clinical decision before assessing the net benefit.

## 5 Conclusion

Preoperative SIRI remained associated with 12-month endoscopic recurrence after ESS following multivariable adjustment, with similar estimates in inverse probability–weighted, multiple-imputation, and AERD-restricted analyses. The SIRI-only model showed moderate discrimination in both cohorts, supporting further evaluation of SIRI as a simple and readily available candidate prognostic marker. Its incremental contribution beyond routine clinical predictors was modest and imprecisely estimated, and the association was attenuated under the deliberately extreme all-missing-as-recurrence scenario. Prospective validation in independent institutions with complete follow-up and prespecified clinical impact evaluation is warranted before SIRI is used to guide treatment or surveillance.

## Data Availability

The participant-level data analyzed in this study are not publicly available because they contain sensitive clinical information and are subject to institutional and ethics restrictions. Requests for access to coded, de-identified data should be directed to Pingcun Wei (wpingcun@126.com) and will be considered subject to approval by Anhui No. 2 Provincial People's Hospital and its ethics committee. Aggregate results and the full model specification are provided in the article and Supplementary Material. The reconstructed analysis and figure code developed for this revision have been retained and may be made available by the corresponding authors upon reasonable request after removal of patient identifiers and local paths. The original executable analysis code is unavailable.
