# Supplementary material for: Preoperative systemic inflammation response index and 12-month endoscopic recurrence after endoscopic sinus surgery in chronic rhinosinusitis with nasal polyps: association and incremental predictive performance in a same-institution, two-campus cohort
Source: Front Med (Lausanne). 2026 Oct 21;13:1983990. doi: 10.3389/fmed.2026.1983990 (PMC13730859; doi:10.3389/fmed.2026.1983990)
Supplement: Supplementary file 1 [file Supplementaryfile1.docx]

# Supplementary Methods

## S1. Cohort integrity and analysis denominators

The analytic dataset comprised 805 coded participant records. The Main Campus development cohort included 445 participants, and the Huangshan Road Campus held-out cohort included 360. The original campus-based allocation was retained without random redistribution. Records were linked and deduplicated across campuses using inpatient medical-record number, national identification number, name, and date of birth; one analytic record per patient was retained, and the final cohorts did not overlap. Because the cohorts arose from different campuses of the same institution and their surgery periods overlapped, the analysis is described as same-institution, different-campus held-out evaluation rather than temporal or external validation.

Primary analyses included participants with an evaluable planned 12-month endpoint: 400 in the development cohort and 320 in the held-out cohort. Participants without an evaluable endpoint (45 and 40, respectively) were retained in cohort descriptions and the attrition analysis but excluded from binary-outcome modelling. The longitudinal dataset contained 2,453 records, including 2,415 planned-visit records and 38 unscheduled assessments. A missing planned visit was treated as missing and not as absence of recurrence.

## S2. Predictors, scaling, and complete-case analysis

Core predictors are age/10; male sex (1 versus female sex 0); asthma (1/0); recorded AERD (asthma plus recorded NSAID allergy, 1/0); number of previous ESS procedures; Lund–Mackay CT/5; total bilateral baseline NPS; blood eosinophils/0.1, with the underlying count in 10⁹/L; and log2(SIRI). The extract does not specify respiratory reactions to NSAIDs or how AERD was clinically confirmed; non-respiratory hypersensitivity may be misclassified. The source Excel formula is SIRI = ROUND(neutrophils×monocytes/lymphocytes, 3), using absolute counts from the same preoperative sample in 10⁹/L. This stored precision is retained for primary reproduction; the unrounded ratio is audit-only. Nonpositive lymphocyte counts or SIRI, missing core predictors, nonbinary categorical inputs, or values outside the clinically valid input ranges must not be silently converted into predictions.

The primary models included all 720 participants with evaluable outcomes and complete values for the model predictors; no predictor imputation was performed. Other descriptive variables were analysed using available observations. Preoperative blood counts were recorded within 0–7 days before surgery. All 805 records indicated no systemic glucocorticoid exposure during the 14 days before blood sampling and no acute infection or fever at baseline.

## S3. Model estimation and performance assessment

The SIRI-only model has an intercept and log2(SIRI). The clinical model has an intercept and the eight routine predictors; the extended model adds log2(SIRI). All models are fitted by maximum likelihood in the development cohort without penalization, class weighting, oversampling, or other imbalance interventions. Full-precision development parameters are used to generate held-out predictions. Table S1 records the model coefficients and coding. The coefficient-scaled equation in the main text is rounded for presentation; numerical reproduction should use the archived full precision.

Wald 95% CIs accompany ORs. AUC estimates and paired comparisons follow the DeLong approach, and Brier scores are the mean squared difference between binary outcome and predicted probability. Calibration-in-the-large is the fitted intercept with LP fixed as an offset; the calibration slope comes from a separate model with a freely fitted intercept and LP coefficient. The freely estimated calibration intercept is not interchangeable with calibration-in-the-large.

For the nested development models, incremental model fit was assessed using a likelihood-ratio test with one degree of freedom. The paired DeLong test was used to compare the fixed clinical- and extended-model predictions in the held-out cohort. The resulting held-out comparison yielded P=0.289. The source development-sample DeLong comparison yielded P=0.375 and is retained only as an exploratory calculation because the nested models were compared in the same data used for fitting.

## S4. Internal bootstrap verification

Internal validation of the extended model used 1,000 nonparametric bootstrap resamples of the development cohort (seed 20260830). In each resample, the same nine-predictor model was refitted and evaluated in both the bootstrap sample and the original development sample. Optimism was estimated as the mean difference between bootstrap-sample and original-sample performance and was subtracted from the apparent estimate. All 1,000 resamples completed successfully. The optimism-corrected AUC was 0.740, the optimism-corrected Brier score was 0.185, and the mean original-sample test calibration slope was 0.874. The latter is a bootstrap test-sample quantity and is distinct from the apparent development calibration slope of 1.

## S5. Thresholds, decision curves, and risk strata

The Youden threshold maximizes sensitivity+specificity−1 on development predictions. Classification used the full-precision development threshold and a probability ≥threshold rule, with the threshold fixed in the held-out cohort. The cutoff was 0.3619492495 when truncated to 10 decimal places for classification-safe display (approximately 0.362). Table S2 gives classification counts and performance; Table S3 reports descriptive risk strata of <20%, 20% to <40%, and ≥40%.

At threshold probability t, net benefit was calculated as TP/n − (FP/n)×t/(1−t). Decision curves for the clinical and extended models were evaluated over thresholds from 0.05 to 0.70 in increments of 0.005 without smoothing. The treat-all strategy used the observed cohort prevalence, whereas the treat-none strategy had a net benefit of 0. The analysis did not incorporate the effectiveness or harms of a specified intervention; therefore, the plotted thresholds are not treatment recommendations.

## S6. Unassessed domains and intended use

No outcome imputation, inverse-probability weighting, subgroup fairness analysis, prospective model-impact study, or formal model-updating analysis was performed. Consequently, the study does not establish performance across demographic or socioeconomic subgroups, the effect of model-guided care, or transportability to other institutions.

The intended potential users are ENT clinicians evaluating preoperative recurrence risk after ESS. The model has not been deployed or evaluated as a clinical system. No clinical action should be assigned to a risk estimate without further external validation and a predefined decision context. Input checks used during statistical verification support research reproducibility but do not constitute a clinically validated software device. No public protocol or prospective registration was available for this retrospective analysis. Patients and the public were not involved in the design, conduct, reporting, or dissemination plans.

## S7. Computational environment and graphical implementation

Analyses were performed using Python 3.12.13, NumPy 2.5.2, pandas 3.0.5, SciPy 1.18.1, statsmodels 0.15.0, and scikit-learn 1.9.0. The bootstrap seed was 20260830.

Calibration plots used ten prediction-quantile groups. The development LOWESS curve was fitted to individual binary outcomes using frac=2/3 and it=0. The held-out logistic calibration curve used a jointly fitted intercept of −0.0247616439 and slope of 1.1854076002. Calibration-in-the-large (−0.1248902407) was estimated separately with the slope fixed at 1. No recalibration was applied to held-out predictions. Decision-curve analysis used thresholds from 0.05 to 0.70 in increments of 0.005 without smoothing or uncertainty bands.

# Supplementary tables

## Supplementary Table S1. Complete logistic model coefficients and predictor coding.

| **Term / coding** | **SIRI-only β** | **Clinical β** | **Extended β** |
| --- | --- | --- | --- |
| Intercept | -0.8680863486 | -4.8720763474 | -4.1085304103 |
| Age (years)/10 | — | 0.1339467685 | 0.1152903153 |
| Male sex (male = 1; female = 0) | — | 0.3457985957 | 0.3502506651 |
| Asthma (yes = 1; no = 0) | — | -0.0819346579 | -0.0598880771 |
| Recorded AERD (yes = 1; no = 0) | — | 0.4559545077 | 0.5744406080 |
| Number of previous ESS procedures | — | -0.1889636919 | -0.2094184357 |
| Lund–Mackay CT score/5 | — | 0.7071107066 | 0.5495208606 |
| Baseline bilateral NPS (0–8) | — | 0.1893590380 | 0.1561007180 |
| Blood eosinophil count (×10⁹/L)/0.1 | — | 0.1791787998 | 0.1319678620 |
| log₂(SIRI); SIRI as stored to 3 decimal places | 0.8442654722 | — | 0.3839203093 |

LP = intercept + sum(β × coded predictor); recurrence probability = 1/[1 + exp(−LP)]. Primary analyses use the supplied SIRI values, calculated from absolute blood-cell counts and rounded to 3 decimal places by the workbook formula. No predictor other than SIRI was log-transformed. Predicted probabilities were calculated before rounding, and coefficients are displayed to 10 decimal places.

## Supplementary Table S2. Classification at the fixed development-derived threshold.

| **Cohort** | **Sensitivity** | **Specificity** | **PPV** | **NPV** | **Accuracy** |
| --- | --- | --- | --- | --- | --- |
| Development | 62.9% | 77.2% | 55.3% | 82.2% | 72.8% |
| Held-out evaluation | 67.7% | 75.1% | 54.9% | 83.8% | 72.8% |

Classification counts at the same fixed threshold:

| **Cohort** | **TP** | **FP** | **FN** | **TN** |
| --- | --- | --- | --- | --- |
| Development | 78 | 63 | 46 | 213 |
| Held-out evaluation | 67 | 55 | 32 | 166 |

A predicted probability at or above the threshold is positive. The development Youden threshold was 0.3619492495 when truncated to 10 decimal places for classification-safe display and was applied unchanged to the held-out cohort. The manuscript shorthand 0.362 should not be used to reproduce the development confusion matrix. This is an exploratory statistical cutoff, not an established treatment threshold. TP, true positive; FP, false positive; FN, false negative; TN, true negative. PPV and NPV are positive and negative predictive values.

## Supplementary Table S3. Observed and predicted recurrence by risk stratum.

| **Cohort** | **Predicted risk** | **n** | **Observed recurrence, n (%)** | **Mean predicted risk** |
| --- | --- | --- | --- | --- |
| Development cohort | <20% | 144 | 14 (9.7) | 12.1% |
| Development cohort | 20–<40% | 137 | 41 (29.9) | 28.4% |
| Development cohort | ≥40% | 119 | 69 (58.0) | 56.9% |
| Held-out cohort | <20% | 100 | 11 (11.0) | 11.9% |
| Held-out cohort | 20–<40% | 120 | 29 (24.2) | 29.6% |
| Held-out cohort | ≥40% | 100 | 59 (59.0) | 58.6% |

Risk strata were <20%, 20% to <40%, and ≥40%. These descriptive cutoffs are not validated intervention thresholds.

## Supplementary Table S4. Evaluable patients and patients without a 12-month endpoint.

| **Variable** | **Evaluable (n = 720)** | **Without endpoint (n = 85)** | **P value** |
| --- | --- | --- | --- |
| Age, years | 47.7 ± 13.0 | 41.6 ± 11.5 | <0.001 |
| Male sex | 402 (55.8) | 47 (55.3) | 0.925 |
| Asthma | 223 (31.0) | 32 (37.6) | 0.211 |
| AERD | 41 (5.7) | 3 (3.5) | 0.612 |
| Baseline NPS | 4.6 ± 1.5 | 4.7 ± 1.5 | 0.531 |
| Lund–Mackay CT score | 12.7 ± 4.6 | 13.2 ± 4.9 | 0.390 |
| Blood eosinophils, ×10⁹/L | 0.24 (0.17, 0.36) | 0.23 (0.16, 0.33) | 0.612 |
| SIRI | 1.006 (0.628, 1.564) | 1.029 (0.654, 1.443) | 0.934 |

Values are mean ± SD, median (25th, 75th percentile), or n (%). Percentages use nonmissing denominators. Two-sided Welch t-tests, Mann–Whitney U tests, or Pearson chi-square tests (Fisher exact when expected counts <5), as appropriate. These descriptive comparisons were not used for predictor selection; P values are unadjusted for multiple comparisons. Patients without a 12-month endpoint were excluded from binary model fitting and validation, not coded as nonrecurrences. These comparisons do not establish that missingness is random.

## Supplementary Table S5. Missingness and recorded treatment information.

| **Field** | **Missing or recorded count** | **Denominator / interpretation** |
| --- | --- | --- |
| Primary model predictors | 0 missing | 805 included records |
| Planned 12-month outcome | 85 missing | 805 included records |
| Baseline SNOT-22 | 7 missing | 805 included records |
| Baseline olfaction VAS | 20 missing | 805 included records |
| CRP | 18 missing | 805 included records |
| Total IgE | 64 missing | 805 included records |
| Histology classification | 18 missing | 805 included records |
| 12-month SNOT-22 | 93 missing | 85 unavailable outcomes plus 8 further missing scores |
| Perioperative systemic glucocorticoids | 505 recorded yes | 805 included records |
| Postoperative intranasal glucocorticoids | 781 recorded yes | 805 included records |
| Postoperative saline irrigation | 777 recorded yes | 805 included records |
| Antibiotics within 7 days before sampling | 28 recorded yes | 805 included records |

# Abbreviations and definitions

| **Abbreviation** | **Definition** |
| --- | --- |
| AERD | Aspirin-exacerbated respiratory disease; operationally recorded as asthma plus NSAID allergy in this dataset |
| AUC | Area under the receiver operating characteristic curve |
| BMI | Body mass index |
| CI | Confidence interval |
| CRP | C-reactive protein |
| CRS | Chronic rhinosinusitis |
| CRSwNP | Chronic rhinosinusitis with nasal polyps |
| CT | Computed tomography |
| DCA | Decision curve analysis |
| ESS | Endoscopic sinus surgery |
| HPF | High-power field |
| IgE | Immunoglobulin E |
| LP | Linear predictor |
| NPS | Nasal polyp score |
| NPV | Negative predictive value |
| NSAID | Non-steroidal anti-inflammatory drug |
| OR | Odds ratio |
| PPV | Positive predictive value |
| ROC | Receiver operating characteristic |
| SD | Standard deviation |
| SIRI | Systemic inflammation response index |
| SNOT-22 | 22-item Sino-Nasal Outcome Test |
| TRIPOD+AI | Transparent Reporting of a multivariable prediction model for Individual Prognosis Or Diagnosis + Artificial Intelligence |

# Supplementary Figure S1

Supplementary Figure S1. Observed recurrence and mean predicted probability by risk stratum. Development and held-out evaluation participants are grouped by extended-model predicted risks <20%, 20% to <40%, and ≥40%. The observed proportions and group mean predictions match Table S3. These exploratory groups are not treatment recommendations.
