# Supplementary material for: Preoperative systemic inflammation response index and 12-month endoscopic recurrence after endoscopic sinus surgery in chronic rhinosinusitis with nasal polyps: association and incremental predictive performance in a same-institution, two-campus cohort
Source: Front Med (Lausanne). 2026 Oct 21;13:1983990. doi: 10.3389/fmed.2026.1983990 (PMC13730859; doi:10.3389/fmed.2026.1983990)
Supplement: Supplementary file 2 [file Supplementaryfile2.docx]

**Supplementary Material**

Supplementary Material for "Preoperative systemic inflammation response index and 12-month endoscopic recurrence after endoscopic sinus surgery in chronic rhinosinusitis with nasal polyps: association and incremental predictive performance in a same-institution, two-campus cohort."

# **Supplementary Methods**

## **S1 Cohort integrity and analysis sets**

The analytic dataset comprised 805 unique adults aged at least 18 years; no upper age limit was applied. The Main Campus development cohort contained 445 participants and the Huangshan Road Campus held-out evaluation cohort contained 360. Primary complete-case models used 400 and 320 participants with evaluable 12-month outcomes, respectively. Missing outcomes were never coded as nonrecurrence in the primary analysis and were assigned only in the two explicitly labeled extreme scenarios.

The cohorts were allocated by campus without random redistribution. Both campuses belong to one institutional system, so the second cohort represents same-institution, different-campus held-out evaluation rather than independent-hospital external validation.

## **S2 Predictor definitions and AERD ascertainment**

Core predictors were age/10; male sex; asthma; AERD; previous ESS count; Lund-Mackay CT score/5; total bilateral baseline NPS; blood eosinophils/0.1 x 10^9/L; and log2(SIRI). SIRI was calculated as neutrophils x monocytes / lymphocytes from the same preoperative sample and stored to three decimals.

General NSAID hypersensitivity and AERD were collected separately. AERD required clinician-documented asthma and an upper and/or lower respiratory reaction after aspirin or another cyclooxygenase-1-inhibiting NSAID in a participant with CRSwNP. General cutaneous, gastrointestinal, or nonspecific NSAID reactions alone did not qualify. A uniform study-mandated provocation test was not required, so residual uncertainty from retrospective clinical ascertainment remains.

## **S3 Primary models and performance assessment**

The SIRI-only, clinical, and extended models were fitted by unpenalized maximum likelihood in the development cohort. Development coefficients were applied unchanged to the held-out cohort. Model-specific AUC 95% confidence intervals used DeLong's variance estimator; Brier scores, calibration-in-the-large, and calibration slopes were also reported. No DeLong hypothesis test compared the nested models. Incremental performance was defined as delta AUC = AUC(extended) - AUC(clinical) and Brier-score improvement = Brier(clinical) - Brier(extended), so positive values favor the extended model. No outcome-class weighting, outcome-class resampling, or synthetic oversampling was used.

Paired uncertainty used 2,000 nonparametric bootstrap replicates. Development and held-out participants were independently resampled in each replicate; both nested models were refitted in the development resample and evaluated pairwise in the development and held-out resamples. Ninety-five-percent percentile intervals used the 2.5th and 97.5th percentiles of the bootstrap distribution. Reclassification indices, decision-curve analysis, the Youden threshold, and post hoc risk strata were not used because no clinical action or defensible action threshold was prespecified.

## **S4 Missing-outcome analyses**

Baseline imbalance by endpoint availability was summarized with standardized mean differences. Fixed clinical- and extended-model predictions were compared between evaluable and nonevaluable participants without using the missing outcomes. An observation model was fitted in all 805 participants with endpoint availability as the dependent variable and cohort plus all nine extended-model predictors as covariates. For participants with an observed endpoint, stabilized inverse-probability-of-observation weights were calculated as w = P(R=1 | cohort) / P-hat(R=1 | cohort, X), where R denotes endpoint availability and X denotes the nine predictors. Weights were not truncated. The weighted clinical and extended logistic models were fitted in the 400 evaluable development participants using HC0 robust covariance; their coefficients were applied unchanged to the 320 evaluable held-out participants for performance evaluation. Development and held-out performance metrics in the inverse-probability-weighted analysis were calculated using the corresponding inverse-probability-of-observation weights.

Missing binary outcomes were imputed in 200 proper stochastic imputations, separately by cohort, using all nine baseline predictors. Parameter uncertainty was propagated through multivariate-normal coefficient draws and residual uncertainty through Bernoulli draws. Each completed dataset refitted the clinical and extended models; the SIRI coefficient was pooled with Rubin's rules. Performance values in Supplementary Table S4 are means across the 200 imputed datasets and are not bootstrap confidence intervals. Deterministic extreme scenarios assigned every missing outcome first to no recurrence and then to recurrence.

## **S5 AERD sensitivity analysis**

The AERD sensitivity analysis excluded all participants with recorded NSAID hypersensitivity but without clinically ascertained AERD, while retaining all clinically ascertained AERD cases. This removed 25 participants overall (11 development and 14 held-out), including 23 with evaluable outcomes (9 and 14, respectively). Both models were refitted in the restricted development sample and applied unchanged to the restricted held-out sample.

## **S6 Internal validation and computational details**

Internal validation of the primary extended model used 1,000 ordinary bootstrap resamples of the development cohort (seed 20260830). Optimism-corrected AUC was 0.740, optimism-corrected Brier score was 0.185, and the mean original-sample test calibration slope was 0.874. Paired incremental-performance bootstrap used seed 20260917; all 2,000 replicates completed. Multiple imputation used seed 20260918.

Analyses used Python 3.12.14, NumPy 2.3.5, pandas 2.2.3, SciPy 1.17.0, and scikit-learn 1.8.0. The model is investigational and is intended for validation research rather than current clinical decision-making. No subgroup or fairness analysis, prospective registration, public protocol, patient or public involvement, or user-interaction study was available.

# **Supplementary Tables**

## **Supplementary Table S1 Complete logistic model coefficients and coding**

| **Term and coding** | **SIRI-only beta** | **Clinical beta** | **Extended beta** |
| --- | --- | --- | --- |
| Intercept | -0.8680863486 | -4.8720763474 | -4.1085304103 |
| Age, years/10 | - | 0.1339467685 | 0.1152903153 |
| Male sex, male=1 | - | 0.3457985957 | 0.3502506651 |
| Asthma, yes=1 | - | -0.0819346579 | -0.0598880771 |
| AERD, yes=1 | - | 0.4559545077 | 0.5744406080 |
| Previous ESS procedures | - | -0.1889636919 | -0.2094184357 |
| Lund-Mackay CT/5 | - | 0.7071107066 | 0.5495208606 |
| Baseline bilateral NPS | - | 0.1893590380 | 0.1561007180 |
| Blood eosinophils/0.1 x 10^9/L | - | 0.1791787998 | 0.1319678620 |
| log2(SIRI), per doubling | 0.8442654722 | - | 0.3839203093 |

LP = intercept + sum(beta x coded predictor); recurrence probability = 1/[1 + exp(-LP)]. Full-precision coefficients should be used for numerical reproduction.

## **Supplementary Table S2 Endpoint availability and measured baseline characteristics**

| **Variable** | **Evaluable (n=720)** | **Missing endpoint (n=85)** | **Absolute SMD** |
| --- | --- | --- | --- |
| Age, years | 47.71 ± 12.95 | 41.60 ± 11.51 | 0.499 |
| Male sex | 55.8% | 55.3% | 0.011 |
| Asthma | 31.0% | 37.6% | 0.141 |
| AERD | 5.7% | 3.5% | 0.103 |
| Previous ESS, count | 0.38 ± 0.65 | 0.44 ± 0.66 | 0.092 |
| Lund-Mackay CT score | 12.74 ± 4.65 | 13.22 ± 4.94 | 0.101 |
| Baseline bilateral NPS | 4.63 ± 1.49 | 4.74 ± 1.50 | 0.072 |
| Blood eosinophils, x 10^9/L | 0.29 ± 0.20 | 0.28 ± 0.18 | 0.068 |
| SIRI | 1.21 ± 0.84 | 1.22 ± 0.87 | 0.011 |
| log2(SIRI) | -0.02 ± 0.94 | -0.02 ± 0.95 | 0.008 |
| Held-out cohort | 44.4% | 47.1% | 0.052 |
| Fixed clinical-model predicted risk | 0.320 | 0.311 | 0.048 |
| Fixed extended-model predicted risk | 0.320 | 0.312 | 0.039 |

SMD, standardized mean difference. The predicted-risk comparison uses only baseline predictors and does not reveal the unobserved recurrence status of participants lost to follow-up.

Among 85 participants without an evaluable endpoint, median recorded follow-up was 5.09 months (IQR, 3.71-7.82). Reasons were inability to contact (n=27), voluntary withdrawal (n=19), work or scheduling constraints (n=15), transfer to another institution (n=10), relocation (n=9), and other nonmedical reasons (n=5).

## **Supplementary Table S3 Observation model and stabilized weight diagnostics**

| **Predictor** | **Adjusted OR for endpoint availability (95% CI)** | | **P value** |
| --- | --- | --- | --- |
| Held-out cohort | 0.827 (0.520-1.316) | | 0.423 |
| Age, per 10 years | 1.483 (1.231-1.786) | | <0.001 |
| Male sex | 1.051 (0.660-1.672) | | 0.835 |
| Asthma | 0.628 (0.371-1.064) | | 0.084 |
| Clinically ascertained AERD | 2.070 (0.555-7.714) | | 0.278 |
| Previous ESS, count | 0.921 (0.646-1.313) | | 0.649 |
| Lund-Mackay CT, per 5 points | 0.878 (0.644-1.198) | | 0.414 |
| Baseline bilateral NPS | 0.988 (0.823-1.186) | | 0.900 |
| Blood eosinophils, per 0.1 x10^9/L | 1.073 (0.919-1.254) | | 0.372 |
| log2(SIRI), per doubling | 1.023 (0.749-1.398) | | 0.885 |
| **Weight diagnostic** | **Development** | **Held-out** | **Overall** |
| Mean ± SD | 1.001 ± 0.062 | 0.999 ± 0.068 | 1.000 ± 0.065 |
| Range | 0.917-1.319 | 0.913-1.409 | 0.913-1.409 |
| Effective sample size | 398.5 | 318.5 | 717.0 |
| Truncation | None | None | None |

## **Supplementary Table S4 Missing-outcome sensitivity analyses**

| **Analysis** | **SIRI OR per doubling (95% CI); P** | **Dev delta AUC** | **Held-out delta AUC** | **Dev Brier improvement** | **Held-out Brier improvement** |
| --- | --- | --- | --- | --- | --- |
| Primary complete case | 1.468 (1.057-2.040); 0.022 | 0.007 | 0.010 | 0.004 | 0.004 |
| Stabilized IPW | 1.463 (1.028-2.082); 0.034 | 0.007 | 0.010 | 0.004 | 0.005 |
| Multiple imputation, m=200 | 1.473 (1.059-2.050); 0.021 | 0.007 | 0.009 | 0.004 | 0.004 |
| All missing=no recurrence | 1.445 (1.053-1.982); 0.022 | 0.005 | 0.009 | 0.003 | 0.003 |
| All missing=recurrence | 1.309 (0.987-1.735); 0.062 | 0.007 | 0.006 | 0.002 | 0.003 |

IPW, inverse-probability-of-observation weighting. Multiple-imputation performance values are means across completed datasets; they are not bootstrap confidence intervals. Delta AUC = extended AUC - clinical AUC; Brier-score improvement = clinical Brier score - extended Brier score; positive values favor the extended model.

## **Supplementary Table S5 AERD sensitivity analysis**

| **Analysis** | **Dev n/events** | **Held-out n/events** | **SIRI OR (95% CI)** | **AERD OR (95% CI)** | **Dev delta AUC** | **Held-out delta AUC** | **Held-out Brier improvement** |
| --- | --- | --- | --- | --- | --- | --- | --- |
| Primary | 400/124 | 320/99 | 1.468 (1.057-2.040) | 1.776 (0.597-5.278) | 0.007 | 0.010 | 0.004 |
| Exclude NSAID hypersensitivity without AERD | 391/120 | 306/96 | 1.461 (1.046-2.041) | 1.796 (0.602-5.354) | 0.006 | 0.008 | 0.004 |

The exclusion removed 25 participants overall, including 23 with evaluable outcomes, while retaining every clinically ascertained AERD case. Delta AUC = extended AUC - clinical AUC; Brier-score improvement = clinical Brier score - extended Brier score; positive values favor the extended model.

## **Supplementary Table S6 Model performance and paired incremental uncertainty**

| **Cohort** | **Model** | **AUC (95% CI)** | **Brier** | **CITL** | **Calibration slope** |
| --- | --- | --- | --- | --- | --- |
| Development | SIRI-only | 0.708 (0.652-0.763) | 0.189 | 0 (apparent) | 1 (apparent) |
| Development | Clinical | 0.757 (0.709-0.805) | 0.179 | 0 (apparent) | 1 (apparent) |
| Development | Extended | 0.764 (0.716-0.812) | 0.175 | 0 (apparent) | 1 (apparent) |
| Held-out | SIRI-only | 0.726 (0.666-0.786) | 0.182 | -0.053 | 1.183 |
| Held-out | Clinical | 0.781 (0.728-0.835) | 0.168 | -0.135 | 1.141 |
| Held-out | Extended | 0.791 (0.737-0.844) | 0.164 | -0.125 (-0.388 to 0.138) | 1.185 (0.869-1.502) |
| **Cohort** | | **Delta AUC extended-clinical (95% bootstrap CI)** | | **Brier improvement clinical-extended (95% bootstrap CI)** | |
| Development | | 0.0073 (-0.0016 to 0.0259) | | 0.0036 (0.00006 to 0.0108) | |
| Held-out | | 0.0096 (-0.0163 to 0.0297) | | 0.0044 (-0.0025 to 0.0111) | |

All 2,000 paired refit-bootstrap replicates completed. Reported 95% intervals are percentile intervals based on the 2.5th and 97.5th percentiles. No hypothesis-test P value was calculated for the nested-model AUC difference. CITL, calibration-in-the-large.

## **Supplementary Table S7 Internal validation of the extended model**

| **Metric** | **Apparent** | **Corrected or mean bootstrap test estimate** |
| --- | --- | --- |
| AUC | 0.764 | 0.740 |
| Brier score | 0.175 | 0.185 |
| Calibration slope | 1.000 | 0.874 (mean original-sample test slope) |

## **Supplementary Table S8 Other missingness and recorded treatment information**

| **Field** | **Missing or recorded count** | **Denominator or interpretation** |
| --- | --- | --- |
| Primary model predictors | 0 missing | 805 included participants |
| Planned 12-month outcome | 85 missing | 805 included participants |
| Baseline SNOT-22 | 7 missing | 805 included participants |
| Baseline olfaction VAS | 20 missing | 805 included participants |
| C-reactive protein | 18 missing | 805 included participants |
| Total immunoglobulin E | 64 missing | 805 included participants |
| Tissue eosinophil classification | 18 missing | 805 included participants |
| 12-month SNOT-22 | 93 missing | 85 unavailable outcomes plus 8 further missing scores |
| Antibiotics within 7 days before blood sampling | 28 recorded yes | 805 included participants |
| Perioperative systemic glucocorticoids | 505 recorded yes | 805 included participants |
| Postoperative intranasal glucocorticoids | 781 recorded yes | 805 included participants |
| Postoperative saline irrigation | 777 recorded yes | 805 included participants |

**Abbreviations**

AERD, aspirin-exacerbated respiratory disease; AUC, area under the receiver operating characteristic curve; CI, confidence interval; CITL, calibration-in-the-large; CRSwNP, chronic rhinosinusitis with nasal polyps; CT, computed tomography; ESS, endoscopic sinus surgery; IPW, inverse-probability weighting; NPS, nasal polyp score; NSAID, non-steroidal anti-inflammatory drug; OR, odds ratio; SIRI, systemic inflammation response index; SNOT-22, 22-item Sino-Nasal Outcome Test.
